# Perioperative Best Practices and Delirium in Patients With Cognitive Impairment

**DOI:** 10.1001/jamanetworkopen.2026.1515

**Published:** 2026-03-02

**Authors:** Danielle Scharp, Kerry Meyers, Girish Nadkarni, Alex Federman, Catherine Sarkisian, Matthew A. Levin, Spencer Perry, Pooja Gownivaripally, Natalia N. Egorova, Yuxia Ouyang, Ira Hofer, Susana Vacas

**Affiliations:** **Author Affiliations:** Division of General Internal Medicine, Icahn School of Medicine at Mount Sinai, New York, New York (Scharp, Federman); Department of AI and Human Health, Icahn School of Medicine at Mount Sinai, New York, New York (Meyers, Nadkarni, Levin, Perry, Gownivaripally); Division of General Internal Medicine and Health Services Research, David Geffen School of Medicine, University California Los Angeles, Los Angeles (Sarkisian); Veterans Administration, Greater Los Angeles, Geriatrics Research Education and Clinical Center, Los Angeles, California (Sarkisian); Department of Anesthesiology, Perioperative and Pain Medicine, Icahn School of Medicine at Mount Sinai, New York, New York (Levin, Hofer); Center for Biostatistics, Department of Population Health Science and Policy, Icahn School of Medicine at Mount Sinai, New York, New York (Egorova, Ouyang); Department of Anesthesiology, Mass General Brigham, Harvard Medical School, Boston, Massachusetts (Vacas).

## Abstract

**IMPORTANCE:**

Despite substantial advances in perioperative care, patients with cognitive impairment are at increased risk for postoperative delirium. Understanding the association of perioperative best practices with delirium prevention may inform tailored strategies for this high-risk group.

**OBJECTIVE:**

To examine associations between anesthesia-related best practices and postoperative delirium in patients with cognitive impairment.

**DESIGN, SETTING, AND PARTICIPANTS:**

This observational cohort study involved a secondary, exploratory analysis of a randomized clinical trial of a clinical decision support system designed to identify patients with cognitive impairment and promote adherence to best practices. The study was conducted between 2023 and 2024 at perioperative areas at a large, urban, academic medical center in New York. Participants were adults scheduled for surgery with preoperative cognitive impairment who stayed in the hospital for at least 1 postoperative night and underwent delirium assessment. Patients undergoing organ donation surgery were excluded.

**EXPOSURES:**

Twelve perioperative best practices across 5 domains were selected and tracked, reflecting avoidance of potentially inappropriate medications, perioperative glycemic control, hemodynamic management, normothermia maintenance, and anesthetic monitoring.

**MAIN OUTCOMES AND MEASURES:**

The primary outcome was postoperative delirium assessed by the 4 A’s Test. Associations between best practices and postoperative delirium were assessed using logistic regression models.

**RESULTS:**

The study included 1255 patients with cognitive impairment (mean [SD] age, 65.0 [15.1] years; 652 male [52%]), and 426 (33.9%; 95% CI, 31.4%-36.6%) developed delirium. Best practices, such as maintaining glucose less than 200 mg/dL (adjusted odds ratio [aOR], 0.41; 95% CI, 0.24-0.69; *P* = .001), using a temperature probe (aOR, 0.65; 95% CI, 0.45-0.96; *P* = .03), and maintaining temperature greater than 36 °C (aOR, 0.64; 95% CI, 0.43-0.94; *P* = .03), were associated with reduced delirium odds in separate models. No individual practice remained significant when analyzed concurrently, apart from postoperative glucose monitoring (aOR, 1.54; 95% CI, 1.11-2.13; *P* = .009).

**CONCLUSIONS AND RELEVANCE:**

This cohort study of surgical patients with cognitive impairment found that current perioperative strategies derived from established recommendations, while foundational, were insufficient to prevent postoperative delirium. The high rate of postoperative delirium highlights the need to refine perioperative care pathways and identify novel strategies that are most effective for cognitively vulnerable populations. Tailored interventions that integrate electronic health record–derived perioperative data and scalable technological tools in anesthesia workflows can help identify and manage high-risk populations.

## Introduction

Over 1 million older adults with cognitive impairment undergo surgical procedures in the US annually.^1^ Alarmingly, up to 50% of these patients experience delirium, marked by altered levels of consciousness, attention, and thought processes that may persist for upto1 week postoperatively.^2-4^ Postoperative delirium is associated with several adverse outcomes, including functional decline,^5^ prolonged hospitalization,^6^ increased risk of institutionalization,^7^ accelerated cognitive impairment, and, ultimately, dementia.^8^ Postoperative delirium–related complications are estimated to contribute to over $32 billion in annual health care costs in the US alone.^9^

Although surgery is often unavoidable, evidence suggests that approximately 40% of postoperative delirium cases may be preventable.^10^ Comprehensive systems of care that implement multicomponent nonpharmacological approaches can mitigate risk.^10,11^ Simple to implement preventive strategies include perioperative cognitive screening and optimization of modifiable risk factors, such as blood pressure,^12^ blood glucose,^13^ body temperature,^14^ anesthetic depth,^15^ and avoidance of potentially inappropriate medications.^16,17^ These elements are included in the American Geriatrics Society and American College of Surgeons Best Practices Statement and the American Society of Anesthesiologists (ASA) recommendations.^17-22^ Despite these evidence-based guidelines, adherence to recommended best practices remains inconsistent in clinical settings,^23,24^ and the incidence of delirium remains high.^25^ In addition, although cognitive impairment is a well-established risk factor for postoperative delirium, preoperative cognitive screening is infrequently performed, with reported rates below 15% in large surgical cohorts.^23,24^ This gap in identification may impede the efficient recognition of high-risk patients and the delivery of personalized care aligned with best practices.^25^

We aimed to examine associations between anesthesia-related best practices and postoperative delirium in patients with cognitive impairment. This approach allowed us to leverage a cohort at high risk for postoperative delirium and to evaluate the independent impact of each practice. As a primary end point, we report the association of each practice with postoperative delirium as determined from a multivariable model to account for interactions among the best practices. Given that the pathophysiology of postoperative delirium is not fully understood and many perioperative recommendations are based on expert consensus, evaluating these practices individually may help clarify their relevance and guide more effective prevention strategies in high-risk surgical populations.

## Methods

### Study Design

This cohort study was conducted in accordance with all regulatory requirements and ethical principles for human participant research, including the World Medical Association Declaration of Helsinki on Ethical Principles for Medical Research Involving Human Subjects,^26^ and was reviewed by Advarra institutional review board (IRB). Approval of transfer of oversight was obtained from the Icahn School of Medicine at Mount Sinai IRB, which is the local IRB where the study took place. Approval and waiver of consent were obtained for this study. This study was conducted in accordance with the Strengthening the Reporting of Observational Studies in Epidemiology (STROBE) reporting guidelines.

We conducted a secondary, exploratory analysis of data from a randomized clinical trial (RCT)^27^ that evaluated the effectiveness of a clinical decision support system in promoting adherence to best practices aimed at decreasing postoperative delirium among patients with cognitive impairment at The Mount Sinai Hospital, New York, New York. Patients aged 18 years or older scheduled for surgery were screened preoperatively by a validated natural language processing algorithm designed to identify cognitive impairment. Patients were included if they stayed in the hospital for at least 1 postoperative night and underwent formal delirium assessment. For patients with multiple surgical procedures during the study period, only the first surgery was included. Exclusion criteria were surgical procedures performed for organ donation. In the intervention group, the clinical decision support system delivered real-time prompts within the electronic health record (EHR) sidebar to anesthesia teams, highlighting patients at high risk for delirium and recommending adherence to best practices. Enrollment for the parent trial^27^ started on June 20, 2023, and terminated on August 31, 2024, due to low enrollment.

For the current study, data from the intervention and control groups were pooled to examine relationships between best practice adherence and postoperative delirium, rather than to compare the associations of the clinical decision support system. Sample characteristics by randomization group were similar (eTable 1 in Supplement 1), supporting pooling data for this analysis. Data were extracted from The Mount Sinai Hospital’s EHRs using a perioperative data warehouse platform.^28,29^ Study variables and operational definitions, including best practices, are provided in eTable 2 in Supplement 1.

### Identification of Patients With Preoperative Cognitive Impairment

The parent RCT^27^ used a natural language processing algorithm trained on a cohort of patients labeled for cognitive impairment confirmed bya comprehensive neurological examination. The algorithm was trained on free-text clinical notes from the EHRs and validated in a subset of surgical patients. The final study cohort included patients identified by the natural language processing algorithm, those with a previously documented Mini-Mental State Examination score 23 or less, and relevant *International Classification of Diseases, Ninth Revision (ICD-9)* and *International Statistical Classification of Diseases and Related Health Problems, Tenth Revision (ICD-10)* codes for cognitive impairment and dementia (eTable 3 in Supplement 1).^30,31^ Although this approach relied on EHR documentation, combining structured and unstructured data sources enabled robust case identification.

### Independent Variables: Anesthesia Perioperative Best Practices

Perioperative best practices were selected on the basis of recommendations from the American Geriatrics Society,^19^ the American College of Surgeons,^18^ and the ASA,^17^ as well as clinical domain expertise. Twelve best practices were identified according to feasibility for consistent monitoring and data extraction: avoidance of diphenhydramine, scopolamine, and midazolam; preoperative glucose assessment; intraoperative glucose monitoring every 2 hours; maintenance of glucose less than 200 mg/dL; postoperative glucose assessment; maintenance of mean arterial pressure greater than 65 mm Hg; use of a temperature probe, maintenance of temperature greater than 36 °C, maintenance of age-adjusted mean alveolar concentration less than 1, and intraoperative use of a processed electroencephalogram to titrate the anesthetic depth. We created binary variables for each best practice, indicating whether the anesthesia provider implemented the practice or not.

### Covariates

Covariates were selected a priori according to their potential to confound the association between best practices and postoperative delirium, informed by prior literature.^32,33^ The following covariates were included: age, sex, race (Asian or Pacific Islander, Black, White, other [Native American or >1 race], and unknown), ethnicity (Hispanic, non-Hispanic, and unknown), body mass index (BMI; calculated as weight in kilograms divided by height in meters squared), ASA physical status score, surgical service, type of anesthesia, duration of anesthesia, and randomization group. Race and ethnicity were included because prior studies have demonstrated associations with differences in perioperative care processes and postoperative outcomes.^34^

### Outcome

Postoperative delirium was defined as a score 4 or higher on the 4 A’s Test (4AT) administered by trained personnel after completion of the recovery phase (discharge from postanesthetic recovery unit), within 7 days following surgery.^35,36^ The 4AT has high validity and reliability for delirium screening, with 95.5% (95% CI, 77.2%-99.9%) sensitivity, 99.2% (95% CI, 98.1%-99.8%) specificity, and area under the curve of 0.998 (95% CI, 0.995-1.000) in the surgical setting.^35,36^ A binary outcome variable was created to indicate whether a patient developed delirium or not. All 4AT scores were extracted from the EHR flowsheet.

### Statistical Analysis

Descriptive statistics (means, SDs, frequencies, and percentages) were used to summarize the study sample. With an α level set at .05, we performed analyses using *t* tests to compare continuous variables and χ^2^ tests to compare categorical variables to examine differences in sample characteristics by delirium status. To examine associations between adherence to best practices and postoperative delirium, we constructed a series of logistic regression models. All models were specified as mixed-effects logistic regressions with a random intercept for 90 attending anesthesiologists to account for potential clustering at the practitioner level. We built a baseline model to estimate the odds of postoperative delirium as a function of sociodemographic and clinical covariates, including age, sex, race, ethnicity, BMI, ASA physical status score, surgical service, type of anesthetic, duration of anesthesia, and randomization group. Next, we used separate logistic regression models, each adding 1 best practice to the baseline model, to evaluate its individual association with delirium. Finally, we used a multivariable logistic regression model that included all best practices together, adjusting for the same set of covariates. Given the exploratory nature of this secondary analysis, formal correction for multiple comparisons was not applied. All analyses were performed using R statistical software version 4.4.2 (R Project for Statistical Computing).

## Results

### Sample Characteristics

Of 4587 eligible patients with preoperative cognitive impairment and a hospital stay of at least 1 night, the final sample included 1255 surgical patients who had delirium assessed with the 4AT (mean [SD] age, 65.0 [15.1] years; 652 male [52%]). Postoperative delirium occurred in 426 participants (33.9%; 95% CI, 31.4%-36.6%). Affected patients were significantly older than those who were not affected (mean [SD] age, 67.0 [15.2] vs 64.0 [15.0] years). ASA physical status score differed significantly by delirium status, with a higher proportion of patients with delirium having an ASA score of3 or higher compared with those without delirium (377 patients [89.0%] vs 701 patients [84.7%]). Duration of anesthesia was significantly longer among patients who developed delirium vs those who did not (mean [SD], 221 [195] vs 183 [161] minutes). Additional sample characteristics are provided in Table 1 and eTable 4 in Supplement 1. The Figure presents the study flowchart, showing enrollment, exclusions, and final analytic sample.

### Associations Between Best Practice Adherence and Postoperative Delirium

For most of the 1255 surgical procedures, diphenhydramine (1233 procedures [98.2%]) and scopolamine (1254 procedures [99.9%]) were avoided, mean arterial pressure was maintained greater than 65 mm Hg (1254 procedures [99.9%]), glucose levels were maintained less than 200 mg/dL (1138 procedures [90.5%]), temperature was maintained greater than 36 °C (1089 procedures [86.6%]), age-adjusted mean alveolar concentration was maintained less than 1 (684 procedures [54.4%]), and a temperature probe was used (1076 procedures [85.6%]). Statistically significant differences were observed between the delirium and no delirium groups in best practices related to glucose management. A higher proportion of patients whose glucose was maintained less than 200 mg/dL did not develop postoperative delirium (776 patients [93.4%] vs 362 patients [85.0%]). In contrast, higher proportions of patients whose glucose was monitored every 2 hours (160 patients [37.6%] vs 238 patients [28.8%]) and postoperatively (250 patients [58.7%] vs 373 patients [45.1%]) developed delirium (Table 2).

### Associations Between Best Practices and Postoperative Delirium

Adjusting for age, patient sex, race, ethnicity, BMI, ASA physical status score, surgical service, type of anesthetic, duration of anesthesia, and randomization group, maintaining glucose less than 200 mg/dL (adjusted odds ratio [aOR], 0.41; 95% CI, 0.24-0.69; *P* = .001), using a temperature probe (aOR, 0.65; 95% CI, 0.45-0.96; *P* = .03), and maintaining temperature greater than 36 °C (aOR, 0.64; 95% CI, 0.43-0.94; *P* = .03), were associated with significantly lower odds of postoperative delirium, whereas assessing postoperative glucose was associated with significantly higher odds of postoperative delirium (aOR, 1.55; 95% CI, 1.14-2.12; *P* = .005). In the adjusted model including all 12 best practices together, assessing postoperative glucose remained significant and was associated with higher odds of postoperative delirium (aOR, 1.54; 95% CI, 1.11-2.13; *P* = .009) (Table 3).

## Discussion

In this cohort study, among 1255 surgical patients with preoperative cognitive impairment at a large academic medical center, 33.9% developed postoperative delirium. Adherence to best practices, such as glucose control, temperature monitoring, and normothermia maintenance, was associated with reduced odds of postoperative delirium in separate regression models. When all 12 best practices were included in a single adjusted model, only postoperative glucose assessment retained a significant independent association with delirium.

Best practice adherence was assessed alongside a validated delirium screening tool.^21^ The postoperative delirium rate in our study is higher than that of previous 4AT-based studies, likely reflecting our focus on patients with cognitive impairment.^37^ This enriched sample and prospective design enhanced statistical power to detect associations while avoiding reliance on post hoc delirium estimation. The elevated rate of postoperative delirium in this population emphasizes the urgent need to develop and test strategies tailored to high-risk groups. Although existing literature supports the importance of identifying preoperative cognitive impairment to reduce delirium risk,^38-40^ evidence is mixed regarding the most effective interventions.^22^ Several society advisories recommend multidisciplinary team involvement, including coordinated care from anesthesiologists, surgeons, geriatricians, pharmacists, nurses, and other perioperative teams.^41,42^ Studies suggesting monitoring anesthetic depth and avoidance of perioperative benzodiazepines as the most effective strategies to minimize delirium risk did not focus on patients with preoperative cognitive impairment.^43,44^ Implementing multicomponent, nonpharmacological bundles, such as deprescribing and nutritional supplementation,^22^ cognitive prehabilitation,^45^ sleep optimization,^46,47^ and individualized pain management,^11^ has shown mixed evidence for patients with preoperative cognitive impairment. For example, cognitive prehabilitation has shown inconsistent results, with compliance and severity of baseline cognitive impairment likely influencing efficacy.^45^ In addition, although adequate pain control is associated with lower postoperative delirium rates, evidence for specific nonopioid regimens is less conclusive. These findings highlight the limitations of current generalized prevention strategies in patients with preoperative cognitive impairment and the need for targeted, prospective trials testing multimodal, scalable interventions that address the unique needs of this population. Clinicians should involve cognitively vulnerable adults and caregivers in shared decision-making that considers the neurocognitive and functional implications of surgery and anesthesia.

An unexpected finding was the association between frequent glucose monitoring and a higher incidence of delirium. This likely reflects indication bias, as clinicians may have intensified monitoring for patients they perceived as higher risk. For example, patients with diabetes typically undergo more frequent glucose monitoring, yet abnormal glycemic levels are also associated with postoperative delirium in high-risk patients without diabetes.^48^ In line with current recommendations, this best practice was embedded in the clinical decision support system.^17-19,21,22^ This association may also represent a novel observation, as the association between perioperative glucose testing and delirium has not been extensively studied. Given the exploratory nature of the analysis and the number of comparisons conducted, this finding should be interpreted cautiously and warrants further investigation to determine whether it reflects indication bias, systematic differences in risk, or a true association.

Although several best practices were significant when considered individually, only postoperative glucose retained independent significance in the multivariable model. This likely reflects clinical reality in which multiple best practices are implemented concurrently. Distinct postoperative delirium risk factors may operate through different pathophysiological pathways, suggesting that interventions effective for one subgroup (eg, patients with frailty, advanced age, or cognitive impairment) may not be universally applicable. This study challenges current recommendations for postoperative delirium prevention, which are generalized across populations but may be insufficient for specific high-risk groups. Many perioperative best practices have been established through expert consensus rather than robust clinical trial evidence. Such approaches can lead to premature adoption of interventions that are later found to be ineffective or harmful. Our findings highlight the need for large, pragmatic RCTs to evaluate the efficacy and potential unintended consequences of perioperative best practices, particularly regarding delirium prevention in cognitively vulnerable patients. This represents a paradigm shift toward personalized perioperative care pathways rather than a one-size-fits-all approach. This may be achieved by integrating EHR-derived perioperative data with standardized postoperative delirium assessments to identify patient-specific vulnerabilities and guide future intervention design.

The parent RCT^27^ used a validated natural language processing algorithm to identify patients with preoperative cognitive impairment from free-text clinical notes, addressing a key limitation of structured data and enabling scalable identification of cognitively vulnerable patients. This demonstrates that early identification of patients at risk for delirium can support real-time integration of tailored perioperative care best practices for cognitively vulnerable patients, including those who may not undergo formal cognitive testing. This could help overcome current limitations in risk stratification, streamline preoperative evaluations, and promote targeted postoperative delirium prevention strategies. Understanding these obstacles may inform future technological deployments and help identify the characteristics of successful integration. Future studies should demonstrate that carefully engineered digital tools can improve short-term patient outcomes and implementation metrics, particularly when embedded in complex care settings. These approaches should integrate implementation science principles with user-centered design and participatory engineering, tools routinely used in consumer technology but rarely applied in clinical settings. Embedding best practices into clinical workflows, coupled with multidisciplinary, multimodal interventions tailored to cognitive status, represents a promising direction for enhancing all surgical outcomes.

### Limitations

This study has limitations that should be mentioned. Because of the observational design, we cannot infer causality between adherence to best practices and postoperative delirium. Despite adjusting for a comprehensive set of covariates informed by literature and clinical expertise, residual confounding remains possible. Although we used data from a large academic medical center, the single-center design may limit generalizability to other institutions with different perioperative protocols, patient demographics, or documentation practices. Although cognitive impairment was identified using a natural language processing algorithm supplemented by cognition scores and relevant *ICD-9* and *ICD-10* codes, misclassification remains possible. However, this automated approach offers a scalable and pragmatic framework for identifying cognitively vulnerable patients and informing future integration of other standardized assessments. As this was an exploratory analysis involving multiple comparisons, there is an increased risk of type I error. Although this approach was appropriate for this study’s objective, the findings should be interpreted as hypothesis generating and require confirmation in future studies. Because the cohort inclusion criteria required an overnight stay, our findings may be less generalizable to lower-acuity surgical patients managed with same-day discharge pathways. Although this may introduce selection bias, same-day discharge generally reflects perioperative clinical stability, making overt postoperative delirium less likely at the time of discharge. In addition, delirium screening was incompletely captured in structured documentation, with only 1255 of 4587 eligible patients with preoperative cognitive impairment and a hospital stay of at least 1 night having a documented 4AT assessment. This excluded cohort differed from the study sample across several perioperative characteristics, suggesting nonrandom screening, which may limit applicability to all surgical patients with cognitive impairment. However, unscreened patients were overall healthier, with lower ASA physical status scores, suggesting a lower likelihood of delirium during hospitalization. Nevertheless, the direction of bias remains uncertain given differences across multiple risk-related factors. These findings underscore the need for more consistent implementation and documentation of delirium screening. In addition, although we relied on objective, EHR-extracted data to enhance reliability and clinical relevance, adherence to best practices was assessed using EHR-derived process metrics. These metrics may not fully capture important nuances of clinical decision-making, including clinician intent, timing, or underlying clinical rationale. Nonetheless, the use of objective, routinely collected data enhances the translational relevance and scalability of our findings.

## Conclusions

In this study of 1255 surgical patients with preoperative cognitive impairment, we found that perioperative best practices derived from established recommendations, while foundational, may not sufficiently mitigate delirium among patients with preoperative cognitive impairment. Further research is needed to identify patient-specific vulnerabilities and perioperative strategies that leverage technological tools to enhance precision and scalability. Embedding these tools into perioperative workflows may enable more targeted and efficient protection of cognitively vulnerable patients.

## Supplementary Material

Supplement 1 eTable 1. Demographic, clinical characteristics and adherences to best practice by randomization groups
eTable 2. Study variables, operational definitions, and measurement
eTable 3. List of International Classification of Diseases codes used for the identification of cognitive impairment
eTable 4. Demographic and clinical characteristics of patients who stayed at least one night but did not have a documented postoperative delirium assessment

Supplement 2 Data Sharing Statement

## Figures and Tables

**Figure.**
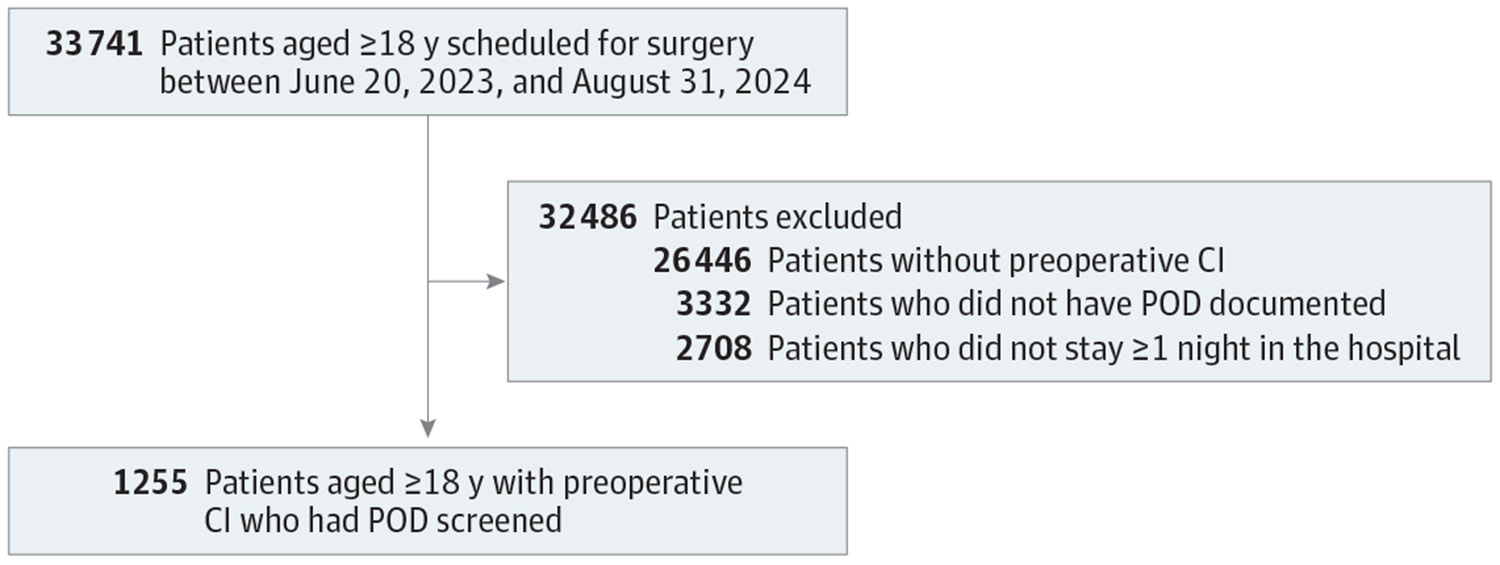
Study Flowchart CI indicates cognitive impairment; and POD, postoperative delirium.

**Table 1.** Demographic and Clinical Characteristics of Patients

Characteristic	Patients, No. (%)			*P* value^a^
	Total (N = 1255)	POD (n = 426)	No POD (n = 829)	
Age, mean (SD), y	65.0 (15.1)	67.0 (15.2)	64.0 (15.0)	.002
Sex				
Female	603 (48.0)	195 (45.8)	408 (49.2)	.27
Male	652 (52.0)	231 (54.2)	421 (50.8)	
Race				
Asian or Pacific Islander	65 (5.2)	17 (4.0)	48 (5.8)	.21
Black	315 (25.1)	115 (27.0)	200 (24.1)	
White	411 (32.7)	126 (29.6)	285 (34.3)	
Other^b^	386 (30.8)	141 (33.1)	245 (29.6)	
Unknown	78 (6.2)	27 (6.3)	51 (6.1)	
Ethnicity				
Hispanic	291 (23.2)	110 (25.8)	181 (21.9)	.29
Non-Hispanic	807 (64.3)	264 (62.0)	543 (65.4)	
Unknown	157 (12.5)	52 (12.2)	105 (12.7)	
Body mass index, mean (SD)^c^	23.3 (12.7)	22.7 (11.9)	23.6 (13.1)	.18
American Society of Anesthesiologists physical status score				
1	5 (0.4)	1 (0.2)	4 (0.5)	<.001
2	78 (6.2)	18 (4.2)	60 (7.2)	
3	563 (44.9)	156 (36.6)	407 (49.2)	
4	499 (39.7)	208 (48.8)	291 (35.1)	
5	16 (1.3)	13 (3.1)	3 (0.4)	
Missing	94 (7.5)	30 (7.0)	64 (7.7)	
Surgical service				
Cardiac	185 (14.7)	75 (17.6)	110 (13.3)	.08
Gastroenterology	252 (20.1)	83 (19.5)	169 (20.4)	
General surgery	84 (6.7)	20 (4.7)	64 (7.7)	
Neurosurgery	101 (8.1)	40 (9.4)	61 (7.5)	
Obstetrics and gynecology	8 (0.6)	3 (0.7)	5 (0.6)	
Orthopedics	49 (3.9)	18 (4.2)	31 (3.7)	
Otolaryngology	18 (1.4)	7 (1.6)	11 (1.3)	
Plastic surgery	13 (1.0)	2 (0.5)	11 (1.3)	
Surgical oncology	3 (0.2)	3 (0.7)	0	
Thoracic	23 (1.8)	7 (1.6)	16 (1.9)	
Urology	13 (1.0)	6 (1.4)	7 (0.8)	
Vascular	158 (12.6)	47 (11.0)	111 (13.4)	
Other	348 (27.7)	115 (27)	233 (28.1)	
Type of anesthetic				
General	696 (55.5)	253 (59.4)	443 (53.5)	.05
Other	559 (44.5)	173 (40.6)	386 (46.5)	
Duration of anesthesia, mean (SD), min	196 (174)	221 (195)	183 (161)	<.001

Abbreviation: POD, postoperative delirium.

a *t* tests were used to compare continuous variables, and χ^2^ tests were used to compare categorical variables, to examine differences in sample characteristics by postoperative delirium status.

b Other race category includes patients whose race was reported as Native American or more than 1 race.

c Body mass index is calculated as weight in kilograms divided by height in meters squared.

**Table 2.** Adherence to Best Practices in Surgical Procedures Performed on Patients With Preoperative Cognitive Impairment

Best practice domain	Patients, No. (%)			*P* value^a^
	Total (N = 1255)	POD (n = 426)	No POD (n = 829)	
Avoid diphenhydramine	1233 (98.2)	419 (98.4)	814 (98.2)	>.99
Avoid scopolamine	1254 (99.9)	426 (100.0)	828 (99.9)	>.99
Avoid midazolam	645 (51.3)	225 (52.8)	420 (50.5)	.47
Assess preoperative glucose	237 (18.9)	92 (21.6)	145 (17.6)	.10
Monitor glucose every 2 h	398 (31.8)	160 (37.6)	238 (28.8)	.002
Maintain glucose <200 mg/dL	1138 (90.5)	362 (85.0)	776 (93.4)	<.001
Assess postoperative glucose	623 (49.7)	250 (58.7)	373 (45.1)	<.001
Maintain mean arterial pressure >65 mm Hg	1254 (99.9)	426 (100.0)	828 (99.9)	>.99
Use temperature probe	1076 (85.6)	356 (83.6)	720 (86.6)	.17
Maintain temperature >36 °C	1089 (86.6)	359 (84.3)	730 (87.8)	.10
Maintain mean alveolar concentration <1	684 (54.4)	217 (50.9)	467 (56.1)	.09
Monitor anesthetic depth	402 (32.1)	147 (34.5)	255 (30.8)	.21

Abbreviation: POD, postoperative delirium.

a χ^2^ tests were used to compare group differences by postoperative delirium status.

**Table 3.** Results of Multivariable Logistic Regression Models Examining Associations Between Anesthesia-Related Best Practices and Postoperative Delirium for Patients With Preoperative Cognitive Impairment

Best practice domain	Unadjusted OR (95% CI)	Adjusted OR (95% CI)^a^	
		Separate model	Combined model
Avoid diphenhydramine	0.87 (0.35-2.18)	0.59 (0.21-1.66)	0.65 (0.22-1.87)
Avoid scopolamine^b^	NA	NA	NA
Avoid midazolam	0.91 (0.71-1.16)	0.86 (0.63-1.18)	0.88 (0.64-1.21)
Assess preoperative glucose	1.29 (0.96-1.74)	1.07 (0.77-1.50)	0.95 (0.67-1.34)
Monitor glucose every 2 h	1.51 (1.16-1.96)	1.18 (0.79-1.77)	1.12 (0.72-1.75)
Maintain glucose <200 mg/dL	0.39 (0.26-0.58)	0.41 (0.24-0.69)	0.57 (0.30-1.06)
Assess postoperative glucose	1.71 (1.34-2.19)	1.55 (1.14-2.12)	1.54 (1.11-2.13)
Maintain mean arterial pressure >65 mm Hg	4.48 (1.35-14.9)	3.74 (0.73-19.08)	2.48 (0.47-13.13)
Use temperature probe	0.77 (0.55-1.07)	0.65 (0.45-0.96)	0.96 (0.23-4.01)
Maintain temperature >36 °C	0.73 (0.52-1.03)	0.64 (0.43-0.94)	0.66 (0.15-2.84)
Maintain mean alveolar concentration <1	0.83 (0.65-1.06)	1.23 (0.79-1.90)	1.27 (0.81-1.98)
Monitor anesthetic depth	1.15 (0.88-1.50)	0.71 (0.47-1.08)	0.72 (0.47-1.13)

Abbreviations: NA, not applicable; OR, odds ratio.

a To evaluate individual associations of each best practice with postoperative delirium, we added 1 best practice to the baseline model in separate models. The combined model included all the best practices together. Models were adjusted for age, patient sex, race, ethnicity, body mass index, American Society of Anesthesiologists physical status score, surgical service, type of anesthetic, duration of anesthesia, and randomization group.

b There were insufficient data to fit the model using avoid scopolamine as the independent variable.

## Data Availability

See Supplement 2.

## Key Points

### Question

Are perioperative best practices derived from established recommendations sufficient to decrease postoperative delirium in patients with cognitive impairment?

### Findings

In this cohort study of 1255 patients, 33.9% developed postoperative delirium. Best practices, such as glucose control and normothermia maintenance, were associated with reduced delirium, although no individual practice remained significant when examined concurrently.

### Meaning

These findings suggest that perioperative best practices may not sufficiently mitigate postoperative delirium, particularly in cognitively vulnerable populations, underscoring the need to refine interventions for high-risk patients.
