# Supplementary material for: Perioperative Best Practices and Delirium in Patients With Cognitive Impairment
Source: JAMA Netw Open. Author manuscript; Available in PMC 2026 Apr 2. (PMC13045776; doi:10.1001/jamanetworkopen.2026.1515)
Supplement: Supplement 1 — eTable 1. Demographic, clinical characteristics and adherences to best practice by randomization groups eTable 2. Study variables, operational definitions, and measurement eTable 3. List of International Classification of Diseases codes used for the identification of cognitive impairment eTable 4. Demographic and clinical characteristics of patients who stayed at least one night but did not have a documented postoperative delirium assessment [file NIHMS2157179-supplement-Supplement_1.pdf]

Supplemental Online Content

Scharp D, Meyers K, Nadkarni G, et al. Perioperative best practices and delirium in patients with cognitive impairment. *JAMA Netw Open*. 2026;9(3):e261515. doi:10.1001/jamanetworkopen.2026.1515

eTable 1. Demographic, clinical characteristics and adherences to best practice by randomization groups

eTable 2. Study variables, operational definitions, and measurement

eTable 3. List of International Classification of Diseases codes used for the identification of cognitive impairment

eTable 4. Demographic and clinical characteristics of patients who stayed at least one night but did not have a documented postoperative delirium assessment

This supplemental material has been provided by the authors to give readers additional information about their work.

© 2026 Scharp D et al. *JAMA Network Open*.

eTable 1. Demographic, clinical characteristics and adherences to best practice by randomization groups.

Characteristic	Total sample (N=1,255) n (%)	Intervention group (n=641) n (%)	Control group (n=614) n (%)	*P* value*
**Age,** mean (SD), years	65 (15.1)	65 (14.9)	65 (15.3)	.59
**Sex**				.89
Female	603 (48)	306 (47.7%)	297 (48.3)	
Male	652 (52)	335 (52.3%)	317 (51.7)	
**Race**				.48
Asian/Pacific Islander	65 (5.2)	37 (5.8)	28 (4.6)	
Black	315 (25.1)	154 (24.0)	161 (26.2)	
White	411 (32.7)	209 (32.6)	202 (32.8)	
Other	386 (30.8)	195 (30.4)	191 (31.2)	
Unknown	78 (6.2)	46 (7.2)	32 (5.2)	
**Ethnicity**				.36
Hispanic	291 (23.2)	155 (24.2)	136 (22.3)	
Non-Hispanic	807 (64.3)	400 (62.4)	407 (66.2)	
Unknown	157 (12.5)	86 (13.4)	71 (11.5)	
**BMI,** mean (SD), kg/m^2	23.3 (12.7)	22.8 (12.3)	23.9 (13.1)	.13
**ASA PS score**				.29
1	5 (0.4)	4 (0.6)	1 (0.2)	
2	78 (6.2)	44 (6.9)	34 (5.5)	
3	563 (44.9)	284 (44.3)	279 (45.5)	
4	499 (39.7)	252 (39.3)	247 (40.2)	
5	16 (1.3)	5 (0.8)	11 (1.8)	
Missing	94 (7.5)	52 (8.1)	42 (6.8)	
**Surgical service**				.73
Cardiac	185 (14.7)	88 (13.7)	97 (15.8)	
Gastroenterology	252 (20.1)	137 (21.4)	115 (18.7)	
General surgery	84 (6.7)	47 (7.3)	37 (6.0)	
Neurosurgery	101 (8.1)	54 (8.4)	47 (7.8)	
OB-GYN	8 (0.6)	3 (0.5)	5 (0.8)	
Orthopedics	49 (3.9)	27 (4.2)	22 (3.6)	
Otolaryngology	18 (1.4)	6 (0.9)	12 (2.0)	
Plastic surgery	13 (1.0)	4 (0.6)	9 (1.5)	
Surgical oncology	3 (0.2)	2 (0.3)	1 (0.2)	
Thoracic	23 (1.8)	12 (1.9)	11 (1.8)	
Urology	13 (1.0)	6 (0.9)	7 (1.1)	
Vascular	158 (12.6)	79 (12.3)	79 (12.8)	
Other	348 (27.7)	176 (27.5)	172 (28.0)	
**Type of anesthetic**				.89
General	696 (55.5)	354 (55.2)	342 (55.8)	
Other	559 (44.5)	287 (44.8)	272 (44.2)	
**Duration of anesthesia,** mean (SD), minutes	196 (174)	193 (174)	198 (175)	.61
**Best practice domain**				
Avoid diphenhydramine	1,233 (98.2)	629 (98.1)	604 (98.4)	.91
Avoid scopolamine	1,254 (99.9)	641 (100)	613 (99.8)	.49
Avoid midazolam	645 (51.3)	331 (51.6)	314 (50.9)	.84
Assess preoperative glucose	237 (18.9)	112 (17.5)	125 (20.5)	.20
Monitor glucose q2h	398 (31.8)	199 (31.0)	199 (32.5)	.62

© 2026 Scharp D et al. *JAMA Network Open*.

Maintain glucose <200 mg/dL	1,138 (90.5)	578 (90.2)	560 (90.9)	.73
Assess postoperative glucose	623 (49.7)	298 (46.5)	325 (50.3)	.02*
Maintain MAP >65 mmHg	1,256 (99.9)	640 (99.8)	616 (100)	>.99
Use temperature probe	1,076 (85.6)	555 (86.6)	521 (84.6)	.35
Maintain temperature >36°C	1,089 (86.6)	561 (87.5)	528 (85.7)	.36
Maintain MAC <1	684 (54.4)	350 (54.6)	332 (54.1)	.92
Monitor anesthetic depth	402 (32.1)	203 (31.7)	201 (32.5)	.79

Note. Bivariate comparisons were conducted to compare differences by randomization group. T-tests were used to compare continuous variables; Chi-square tests were used to compare categorical variables. In the parent randomized controlled trial, in the intervention group, the clinical decision support system delivered prompts within the electronic health record sidebar to anesthesia teams, highlighting patients at high risk for postoperative delirium and recommending adherence to perioperative best practices.

Abbreviations: SD, standard deviation; BMI, body mass index; ASA PS, American Society of Anesthesiologists physical status; q2h, every two hours.

*Statistically significant results

© 2026 Scharp D et al. *JAMA Network Open*.

eTable 2. Study variables, operational definitions, and measurement.

Variable	Operational definition	Measurement
**Independent variables: Anesthesia perioperative best practices**		
Avoid diphenhydramine	Absence of documentation of the administration of diphenhydramine during the preoperative period.	Anesthesia record, EHR
Avoid scopolamine	Absence of documentation of the administration of scopolamine during the preoperative period.	Anesthesia record, EHR
Avoid midazolam	Absence of documentation of the administration of midazolam during the preoperative period.	Anesthesia record, EHR
Check preoperative glucose	Documentation of blood glucose result during the preoperative period.	Anesthesia record, EHR
Check glucose every 2 hours	Documentation of blood glucose results at least every 2 hours during the intraoperative period.	Anesthesia record, EHR
Maintain glucose <200	Absence of documentation of blood glucose readings >200 during the intraoperative period.	Anesthesia record, EHR
Check PACU glucose	Documentation of blood glucose results during the postoperative period.	Anesthesia record, EHR
Keep MAP > 65mmHg	Absence of documentation of MAP <65mmHg during the intraoperative period.	Anesthesia record, EHR
Use temperature probe	Documentation of continuous temperature readings obtained from the temperature probe during the intraoperative period.	Anesthesia record, EHR
Maintain temperature >36°C	Absence of documentation of temperature readings <36°C during the intraoperative period.	Anesthesia record, EHR
Age adjusted MAC <1	Absence of documentation of MAC >1 during the intraoperative period.	Anesthesia record, EHR
Monitor anesthesia depth	Documentation of electroencephalogram-based monitoring with the bispectral index during the intraoperative period.	Anesthesia record, EHR
**Covariates**		

© 2026 Scharp D et al. *JAMA Network Open*.

Age	Age in years at the time of admission for surgery.	EHR flowsheet
Patient sex	Patient sex reported by the patient/documented by the clinician at the time of admission for surgery.	EHR flowsheet
Body mass index	Body mass index at the time of admission for surgery, categorized as: underweight <18.5, standard weight 18.5-29.99, overweight 30-39.99. or obese >40.	EHR flowsheet
Race<br>Ethnicity	Race and ethnicity reported by the patient/documented by the clinician at the time of admission for surgery.	EHR flowsheet
American Society of Anesthesiologists physical status score	The health of a patient as determined by the clinician upon admission for surgery, graded as: 1 – healthy, 2 – mild systemic disease (controlled conditions), 3 – severe systemic disease (uncontrolled conditions), 4 – severe systemic disease with constant threat to life (e.g., severe HF, respiratory disease, recent MI), or 5 – not expected to survive without surgery.	EHR flowsheet
Surgical service	The designated department providing surgery for the patient classified as: cardiac, ear nose and throat, gastroenterology, general surgery, liver transplant, neurology, obstetrics-gynecology, orthopedics, plastic surgery, surgical oncology, thoracic, urology, vascular, or other.	EHR flowsheet
Procedure type	Classification of the surgery based on urgency, setting, or purpose characterized as: elective – non-urgent procedures that can be postponed without immediate risk to the patient’s health, critically emergent – procedures that need to be performed	EHR flowsheet

© 2026 Scharp D et al. *JAMA Network Open*.

	immediately to prevent serious harm or death, emergent – procedures that require prompt attention but are not immediately life-threatening, urgent – procedures that need to be addressed within 24 hours to avoid clinical deterioration of the patient's condition, inpatient – procedures in which the patient is admitted to the hospital for at least one night, or transplant – procedure involving the transfer of an organ or tissue from one body to another.	
Type of anesthetic	The type of anesthetic used during the surgery classified as: general – inhaled gases or intravenous medications that induce complete loss of consciousness, epidural – injection of medication into the epidural space in the spinal cord eliciting numbness of the lower body while the patient remains alert, monitored anesthesia care – local anesthesia with sedation through which the patient is awake but in a relaxed state, regional block – injection of medication near a cluster of nerves to block sensation to a specific area of the body, or spinal – injection of medication into the cerebrospinal fluid of the spinal canal eliciting numbness to the lower body while the patient remains awake.	EHR flowsheet
Duration of anesthesia	Time in minutes during which a patient is under the effects of anesthesia – from administration of the anesthetic medication until the regaining of consciousness.	EHR flowsheet

© 2026 Scharp D et al. *JAMA Network Open*.

**Outcome variable: Postoperative delirium**		
Postoperative delirium	A single positive 4AT score (score ≥4) in the seven days following surgery.	4AT score, EHR flowsheet

Note. Abbreviations: EHR, electronic health record; PACU, post-anesthesia care unit; MAP, mean arterial pressure; MAC, minimum alveolar concentration; POD, postoperative delirium; 4AT, 4 A's Test

© 2026 Scharp D et al. *JAMA Network Open*.

eTable 3. List of International Classification of Diseases codes used for the identification of cognitive impairment.

Category	Code	Meaning
**ICD-9**	290.0	Senile dementia, uncomplicated
	290.1	Presenile dementia
	290.2	Senile dementia with delusional/hallucinatory symptoms
	290.3	Senile dementia with depressive features
	290.4	Arteriosclerotic dementia
	290.8	Other specified dementias
	290.9	Unspecified dementia
	291.1	Alcohol-induced amnestic disorder
	291.2	Alcohol-induced dementia
	292.82	Drug persisting dementia
	294.0	Amnestic syndrome
	294.10	Dementia without behavioral disturbance
	294.11	Dementia with behavioral disturbance
	294.20	Dementia, unspecified
	331.0	Alzheimer's disease
	331.82	Dementia with Lewy bodies
	331.11	Pick's disease (frontotemporal dementia)
	331.19	Other frontotemporal dementia
**ICD-10**	A81.00	Creutzfeldt-Jakob disease, unspecified
	E75.2	Other sphingolipidosis
	E75.23	Krabbe disease
	F01	Vascular dementia
	F02	Dementia in other diseases classified elsewhere
	F0	Unspecified dementia
	F04	Amnestic disorder due to known physiological condition
	F10.26	Alcohol dependence with alcohol-induced persisting amnestic disorder
	F10.27	Alcohol dependence with alcohol-induced persisting dementia
	F10.96	Alcohol use, unspecified with alcohol-induced persisting amnestic disorder
	F10.97	Alcohol use, unspecified with alcohol-induced persisting dementia
	F13.26	Sedative, hypnotic or anxiolytic dependence with persisting amnestic disorder
	F13.27	Sedative, hypnotic or anxiolytic dependence with persisting dementia
	F13.96	Sedative, hypnotic or anxiolytic use, unspecified with persisting amnestic disorder
	F13.97	Sedative, hypnotic or anxiolytic use, unspecified with persisting dementia
	F18.27	Inhalant dependence with inhalant-induced dementia
	F18.97	Inhalant use, unspecified with inhalant-induced persisting dementia
	G10	Huntington's disease
	G20	Parkinson's disease
	G30	Alzheimer's disease
	G23.1	Progressive supranuclear ophthalmoplegia (Steele-Richardson-Olszewski)
	G31.0	Frontotemporal dementia

© 2026 Scharp D et al. *JAMA Network Open*.

G31.85	Corticobasal degeneration
R41.0	Disorientation, unspecified

© 2026 Scharp D et al. *JAMA Network Open*.

eTable 4. Demographic and clinical characteristics of patients who stayed at least one night but did not have a documented postoperative delirium assessment

**Characteristic**	**Total study sample** (N=1,255) n (%)	**No delirium assessment documentation** (n=3,332) n (%)	*P* value
**Age,** mean (SD), years	65 (15.1)	65 (14.5)	>.99
**Sex**			.20
Female	603 (48)	1,674 (50.2)	
Male	652 (52)	1,658 (49.8)	
**Race**			<.001
Asian/Pacific Islander	65 (5.2)	222 (6.7)	
Black	315 (25.1)	799 (24)	
White	411 (32.7)	1,111 (33.3)	
Other	386 (30.8)	1,096 (32.8)	
Unknown	78 (6.2)	104 (3.1)	
**Ethnicity**			<.001
Hispanic	291 (23.2)	897 (26.9)	
Non-Hispanic	807 (64.3)	2,144 (64.3)	
Unknown	157 (12.5)	291 (8.7)	
**BMI,** mean (SD), kg/m^2	23.3 (12.7)	31 (4.0)	<.001
**ASA PS score**			<.001
1	5 (0.4)	7 (0.2)	
2	78 (6.2)	357 (10.7)	
3	563 (44.9)	1,580 (47.4)	
4	499 (39.7)	1,076 (32.3)	
5	16 (1.3)	27 (0.8)	
Missing	94 (7.5)	285 (8.6)	
**Surgical service**			<.001
Cardiac	185 (14.7)	472 (14.2)	
Gastroenterology	252 (20.1)	349 (10.5)	
General surgery	84 (6.7)	302 (9.1)	
Neurosurgery	101 (8.1)	343 (10.3)	
OB-GYN	8 (0.6)	67 (2.0)	
Orthopedics	49 (3.9)	293 (8.8)	
Otolaryngology	18 (1.4)	75 (2.3)	
Plastic surgery	13 (1.0)	50 (1.5)	
Surgical oncology	3 (0.2)	19 (0.6)	
Thoracic	23 (1.8)	85 (2.6)	
Urology	13 (1.0)	135 (4.1)	
Vascular	158 (12.6)	418 (12.5)	
Other	348 (27.7)	724 (21.7)	
**Type of anesthetic**			<.001
General	696 (55.5)	2,256 (67.7)	
Other	559 (44.5)	1,076 (32.3)	
**Duration of anesthesia,** mean (SD), minutes	196 (174)	235 (168)	<.001

Note. The final analytic sample included patients with preoperative cognitive impairment who stayed at least one night in the hospital and had postoperative delirium screened with the 4AT.

© 2026 Scharp D et al. *JAMA Network Open*.

We used t-tests to compare continuous variables and Chi-square test to compare categorical variables to compare sociodemographic and clinical characteristics between the final analytic sample and patients who had preoperative cognitive impairment, stayed at least one night in the hospital, but did not have a documented delirium assessment with the 4 A's Test.

Abbreviations: POD, postoperative delirium; SD, standard deviation; BMI, body mass index; ASA PS, American Society of Anesthesiologists physical status.

© 2026 Scharp D et al. *JAMA Network Open*.
