# Supplementary material for: Perioperative Best Practices and Delirium in Patients With Cognitive Impairment
Source: JAMA Netw Open. Author manuscript; Available in PMC 2026 Apr 2. (PMC13045776; doi:10.1001/jamanetworkopen.2026.1515)
Supplement: Supplement 2 — Data Sharing Statement [file NIHMS2157179-supplement-Supplement_2.pdf]

Data Sharing Statement

Scharp. Perioperative Best Practices and Delirium in Patients With Cognitive Impairment.
JAMA Netw Open. Published March 12, 2026. doi:10.1001/jamanetworkopen.2026.1515

Data
Data available: Yes
Data types: Deidentified participant data, Data dictionary
How to access data: Data can be requested from Ira Hofer at
When available: With publication

Supporting Documents
Document types: None

Additional Information
Who can access the data: Researchers whose proposed use of the data has been approved
Types of analyses: Academic and research purposes, systematic reviews and meta-analyses.
Mechanisms of data availability: Data will be made available with investigator support after approval of a proposal and a signed data access agreement.
